# Rare‐Earth Ion Intercalation in Graphene via Thermal and Electrostatic Control

**DOI:** 10.1002/adma.202502417

**Published:** 2025-07-06

**Authors:** Mengjie Feng, Qing Dai, Anupam Bhattacharya, Ciaran Mullan, Amit Singh, Yangming Fu, Ivan Timokhin, Yanmeng Shi, Alexander Rudnev, Kostya S. Novoselov, Qian Yang, Artem Mishchenko

**Affiliations:** ^1^ Department of Physics and Astronomy University of Manchester Manchester M13 9PL UK; ^2^ National Graphene Institute University of Manchester Manchester M13 9PL UK; ^3^ State Key Laboratory of Semiconductor Physics and Chip Technologies Institute of Semiconductors Chinese Academy of Sciences Beijing 100083 China; ^4^ Center of Materials Science and Optoelectronics Engineering University of Chinese Academy of Sciences Beijing 100049 China; ^5^ Department of Chemistry Biochemistry and Pharmaceutical Sciences University of Bern Bern 3012 Switzerland; ^6^ Institute for Functional Intelligent Materials National University of Singapore Singapore 117544 Singapore

**Keywords:** electrostatic control, graphene, intercalation chemistry, ion intercalation, temperature

## Abstract

Atomic‐scale control and understanding the controlling strategy of ion intercalation are pivotal for advancing energy storage, quantum technologies, and adaptive electronics. While intercalation – the insertion of ions into layered materials – has transformative potential, the mechanisms driving it, particularly for rare‐earth ions, remain poorly understood. Here, a thermal‐electrostatic strategy is developed to achieve reversible and tunable europium ion intercalation that enables precise control over intercalation dynamics. This study investigates how temperature and voltage influence the intercalation of europium ions into bilayer graphene. Our results reveal the formation of a 2D europium layer and ionic state of intercalation europium within the graphene structure, providing fundamental insights into intercalation energetics. This work establishes a versatile platform for designing adaptive 2D heterostructure, engineering advanced materials and devices with unique electronic and optoelectronic properties.

## Graphical abstract

Europium ions reversibly intercalate into the van der Waals gap of bilayer graphene when heat and electrostatic gate act together, switching the stacking order and electronic response on demand. Real‐time transport, simulations, and device tests expose a hidden 2D Eu sheet and its temperature‐tuned diffusion, showing a versatile electrochemical route toward reconfigurable 2D heterostructures with rare‐earth functionality.

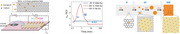

Intercalation,^[^ ^1^, ^2^, ^3^ ^]^ especially metal ion intercalation, is critical for the efficient operation of energy storage,^[^ ^4^, ^5^ ^]^ manipulating materials properties,^[^ ^6^, ^7^, ^8^ ^]^ and engineering materials with wide‐ranging applications, from biosensing to quantum technologies.^[^ ^9^, ^10^, ^11^, ^12^, ^13^ ^]^ The intercalation of layered crystals enables modification of both chemical^[^ ^14^ ^]^ and crystallographic^[^ ^15^ ^]^ properties, allowing for the synthesis of novel atomically thin materials^[^ ^16^, ^17^, ^18^ ^]^ and heterostructures.^[^ ^19^, ^20^ ^]^ Despite its significance for relevant applications in energy storage and conversion,^[^ ^21^, ^22^, ^23^ ^]^ biochemical processes,^[^ ^9^ ^]^ on‐chip inductors,^[^ ^10^ ^]^ superconductivity,^[^ ^11^ ^]^ and ferromagnetism,^[^ ^12^ ^]^ the mechanisms of electrochemical intercalation^[^ ^24^, ^25^, ^26^, ^27^ ^]^ and the ionic state of intercalants^[^ ^28^, ^29^ ^]^ is rarely experimentally verified.

2D materials and their heterostructures^[^ ^30^, ^31^ ^]^ provide a highly tunable platform to explore and control electrochemical intercalation, diffusion, and crystallisation of metal ions within van der Waals (vdW) interlayer.^[^ ^32^ ^]^ Previous work on metal electrochemical intercalation in few‐layer graphene mainly focuses on Li and other alkali metal ion intercalation,^[^ ^29^, ^32^, ^33^, ^34^, ^35^ ^]^ demonstrating understanding of the intercalation processes including structure of the metal intercalant layer, ion diffusion, and optical properties of the intercalation compound. Intercalation of alkali metal ions have also been attempted between mono/bilayer graphene and substrates. These studies have revealed that the mechanism includes the introduction of the intercalant through a defect in graphene or the substrate.^[^ ^16^, ^36^ ^]^ The intercalation of rare earth metals like Eu, Gd or Dy between graphene and substrates such as SiC have shown a different mechanism than alkali metals. Deposited rare‐earth atoms diffuse inside graphene layers with high temperature annealing.^[^ ^37^, ^38^ ^]^ All these studies on intercalation within or below graphene are comparatively recent compared to intercalation of rare earth atoms inside graphite interlayers, which was attempted almost 70 years back.^[^ ^39^, ^40^ ^]^ These studies were originally aimed at modulating magnetic properties with the introduction of rare earth metal Eu, and other rare earth metals share large similarities with alkali metals in ion radii and electronegativity, whose intercalation between graphene and the substrate has also been investigated^[^ ^37^, ^38^, ^39^, ^40^, ^41^, ^42^, ^43^, ^44^, ^45^ ^]^ Like the alkali metals and alkaline earth metals, Eu forms EuC_6,_ leading to + 2 oxidation state. We also see that the intercalation of Eu in graphite expands the interlayer space significantly. On the other hand, in contrast with alkali metals, the large mass of Eu limits its dynamics, which is why the intercalation of Eu into graphite interlayers could take up to 20 days at 520 °C.^[^ ^46^, ^47^ ^]^ making it a prohibitive process in practice. In this work, we demonstrate a tunable and reversible intercalation of rare earth metal europium (Eu) into the vdW gap of mechanically exfoliated bilayer graphene (BLG) at much lower temperature (≈155 °C), using a combination of electrochemical priming, thermally activated diffusion, and electrostatic gating to control the intercalation process. This procedure decouples the electrochemical reaction step from the intercalation step, enabling a clearer understanding of the roles of the two most critical factors in electrochemical intercalation: temperature and voltage. Our work introduces a straightforward and fast approach for the reversible intercalation of multivalent rare‐earth metals into 2D materials, and opens new avenues for engineering graphene‐based devices, with significant potential applications in intercalation chemistry, high‐performance electronics, quantum information processing, and adaptive optoelectronic technologies.

## 1 Europium Electrochemical Intercalation and Layer Formation at High Temperature

**Figure 1:**
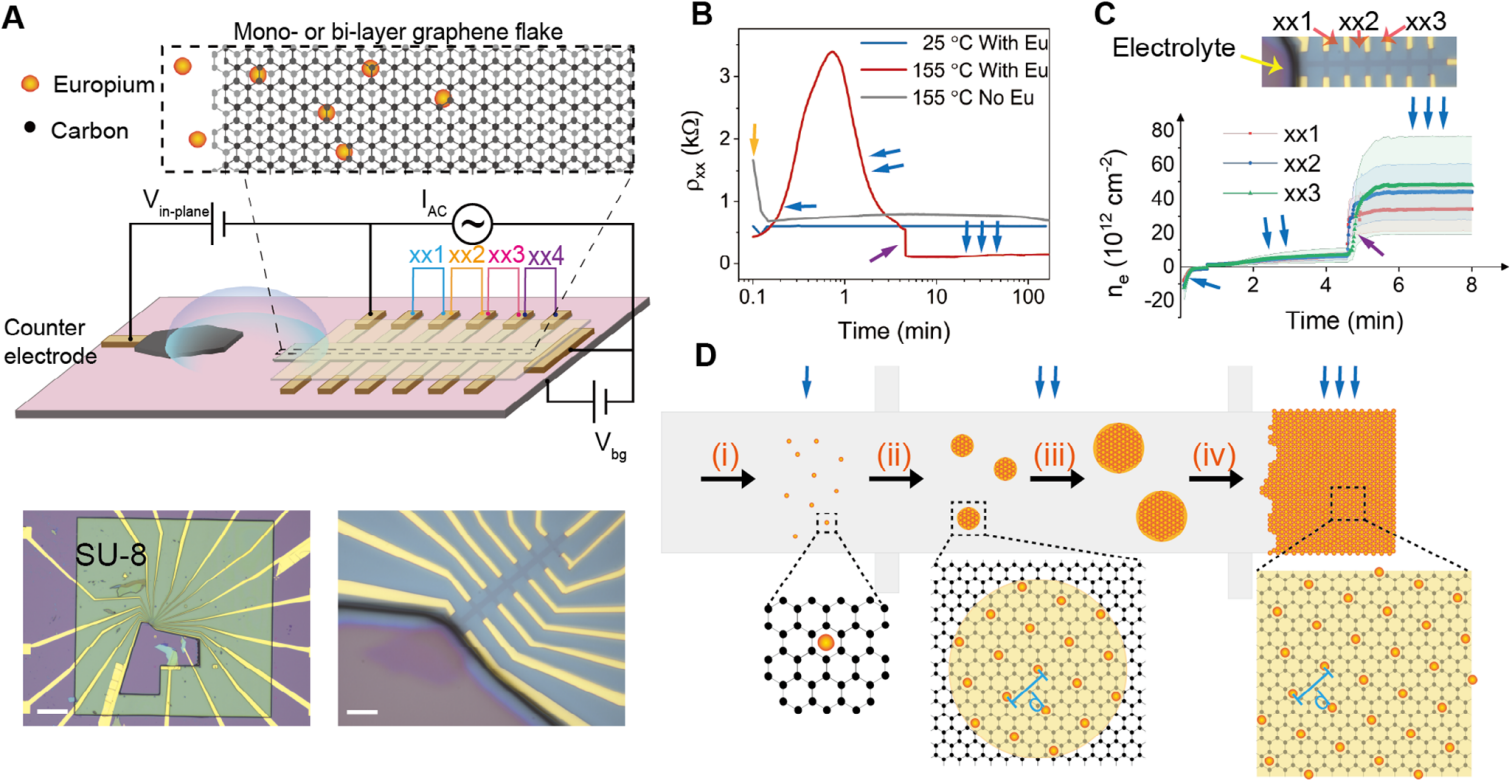
Eu intercalation and Eu 2D layer formation. A) Schematic and optical micrographs of one of our devices (device 1). Labels xxi (i = 1, 2, 3, 4) are neighbouring positions on the Hall bar with i = 1 being closest to the electrolyte. Scale bars of optical micrographs, left: 200 µm, right: 10 um. B) Time dependence of longitudinal resistivity *ρ*_xx_ of BLG intercalated with Eu at 25 °C (blue, xx1) and 155 °C (red, xx2), and reference sample without Eu at 155 °C (gray, xx2). The electrochemical reaction driving force V_in‐plane_ = 8 V is applied from *t* = 0.1 min. C) Time dependence of qualitatively estimated charge carrier density transferred from intercalant, extracted from *ρ*_xx_(*t*) with error margins shown in coloured background. Insert: Optical micrograph of BLG Hall bar device 1. D) Schematic of the intercalation process and the formation of Eu 2D layer. Three insets show the progression of the intercalation process; the interatomic Eu‐Eu distance *d* is also labeled.

We performed temperature‐controlled intercalation using an experimental setup similar to those used for lithium‐ion intercalation.^[^ ^27^, ^32^ ^]^ The device fabrication and intercalation process are detailed in Materials and Methods (Supporting Information). In brief, BLG flakes exfoliated onto SiO_2_/Si substrate are patterned into Hall bar shape and electrically contacted via gold electrodes. SU‐8 photoresist is used to partially isolate BLG and fully isolate electrodes from the electrolyte so that intercalation can only take place where graphene is in direct contact with the electrolyte (see Materials and Methods, Supporting Information “Device fabrication” and **Figure** **1**a). Two electrolytes are used: one is Eu‐containing polyethylene oxide (PEO) electrolyte, the other is pure PEO without Eu. In a typical experiment, one end of a multiterminal BLG device is exposed to electrolyte. When the temperature is raised to 155 °C with an in‐plane bias *V*_in‐plane_ = 8 V applied between the BLG and graphite counter electrode, intercalation is initiated (see Materials and Methods, Supporting Information “Electrochemical intercalation”). The thick SU‐8 protective layer on top of the device prevents common characterization methods, such as atomic force microscope and tunnelling electron microscope, used to visualize the intercalants. Moreover, removing the encapsulation leads to delamination and rapid oxidation of the Eu interlayer. Therefore, to monitor the intercalation process, we instead resort to the longitudinal resistivity of BLG as a function of time, *ρ*_xx_(*t*) (see Materials and Methods, Supporting Information Electronic transport measurements).

We optimized the temperature of intercalation by carrying out the experiments at various temperature T = 70, 80, 130, 140, 155, and 185 °C. We find that intercalation begins at 140 °C but remains slow. At 185 °C the initial intercalation is rapid but the conductivity later decays as the polymer starts to degrade. This is in agreement with reported thermogravimetric studies on PEO that show dissociation of PEO near 200 °C.^[^ ^48^, ^49^ ^]^ We therefore chose 155 °C as the optimum: it is high enough to overcome the diffusion barrier yet still comfortably below the onset of PEO degradation. See the Materials and Methods (Supporting Information) Temperature control of Eu intercalation and Figures S3 and S7 (Supporting Information) for further details.

When the electrolyte is in contact with the BLG device at 155 °C, and *V*_in‐plane_ = 8V is applied, a large transient current passes through the device, evidenced by the low resistivity in *ρ*_xx_ at *t* ≈ 0.1 min (red curve at the yellow arrow in Figure 1b; Figure S1a, Supporting Information). In the case of Eu containing electrolyte, *ρ*_xx_(*t*) increases and peaks at ≈3.5 kΩ at *t* ≈ 1 min, before falling rapidly to ≈100 Ω at *t* ≈ 5 min and remaining nearly constant afterward. The peak in *ρ*_xx_(*t*) is attributed to the change in the doping of BLG from p‐type to n‐type. BLG is p‐doped before intercalation from the proximity of SU‐8 (single blue arrow in Figure 1b,c) and becomes n‐doped after intercalation of Eu ions which leads to rise in electron density in graphene to balance the positive charge of intercalated Eu (see Figure S1c, Supporting Information and double blue arrow in Figure 1b,c). The observed strong electron doping of BLG agrees with our Bader charge analysis and estimated density of intercalated Eu (see Materials and Methods, Supporting Information “Computation”). Results from two control experiments (carried out at *T* = 25 °C with Eu‐containing electrolyte, and at T = 155 °C with pure PEO electrolyte) are plotted in Figure 1b and Figure S1a (Supporting Information). No notable change in *ρ*_xx_(*t*) is observed at room‐temperature. The control experiment at high temperatures with pure PEO electrolyte also indicates that PEO does not intercalate into BLG, even at an elevated temperature of 155 °C.

The measured *ρ*_xx_(*t*) of the Eu‐BLG system drops to ≈100Ω at *t* = 5 min and remains nearly constant afterward, which pinpoints the end of intercalation and concomitant phase transition (see also the drop in *ρ*_xx_ in Figure S1a, Supporting Information). Based on the results in Figure 1b, we qualitatively estimated electron density in BLG as, *n_e_* = 1/*ρ_xx_eμ_0_* (where *μ_0_* is the mobility at the beginning of intercalation, see also Materials and Methods, (Supporting Information) “Electronic transport measurements” for more details) during Eu intercalation as a function of time and plotted in Figure 1c. The sheer increase in the electron density at *t* ≈ 5 min (purple arrow in Figure 1c) and the constant value afterward (triple blue arrow in Figure 1c) indicate the successful intercalation of Eu between BLG and the intercalated Eu concentration remains constant. Our estimated charge carrier density of intercalated BLG using Equation (1) is *n_e_* = (3.7 ± 0.6) × 10^14^cm^−2^ at t = 5 min, which is close to the charge carrier density, *n_Hall_* ≈ 4.1 × 10^14^cm^−2^, extracted from Hall effect measurements after intercalation (Figure S2c, Supporting Information).

We propose the following mechanism of the Eu layer formation in BLG during electrochemical intercalation as schematically shown in Figure 1d: (stage i) Eu ions enter BLG interlayer, diffuse, and randomly bind to free interlayer sites, (stage ii, iii) bound Eu ions further attract mobile Eu ions to its surrounding to form islands of Eu crystallites, continuing growth of Eu crystallites (we considered two structures, C_6_EuC_6_ with a lattice constants *a* = *b* = 4.28Å and *γ* = 120°, and C_14_EuC_14_ with *a* = *b* = 6.52Å and *γ* = 120°; see Materials and Methods, Supporting Information “Computation”) (stage iv) expanding crystallites interconnect to fill in the BLG interlayer. The linear rise of *n_e_* (marked as double blue arrow in Figure 1c) marks the stage i–iii when Eu^3+^ ions from electrolyte come into contact with the negatively charged BLG electrode and are reduced because of the electronegativity difference between Eu and C (this leads to an increase in electron density of BLG as it is electrically grounded) to form clusters. As Eu ions diffuse into the BLG interlayer, the intercalation rate slows down, because more Eu ions can only intercalate when pervious ions diffuse deeper into the BLG interlayer. Simultaneously, the electron mobility decreases because of the increased impurity scattering. This agrees with the observed slowdown in the slope of *n_e_* marked with a double blue arrow in Figure 1. We model the relation between the rate of intercalation and charge carrier density *n_e_* in graphene during intercalation stages i–iii using a model of crystal growth under long‐range diffusion^[^ ^50^, ^51^ ^]^

(1) $$n_{e}=\frac{z\pi v\left(t-t_{0}\right)}{A_{\mathrm{Eu}}}\;$$

here, *A*_Eu_ = *πd* ^2^sin*γ* is the unit cell area of Eu 2D crystal, *t_0_* is the growth start time, *z* is the charge transferred from a single intercalated Eu ion to BLG, and the average intercalation rate is *v*. At the beginning of the intercalation process, due to low intercalated Eu concentration, we assume that the electron mobility of intercalation compound in this stage remains unchanged from the initial mobility. By fitting Equation (1) to *n_e_* (*t*) curves in the range of t ≤ 0.25 min (Figure 1c; Figure S1b, Supporting Information) we obtain *v* = 0.066 ± 0.010 min^−1^ for C_14_EuC_14_ and 0.028 ± 0.004 min^−1^ for C_6_EuC_6_. We observe from Figure 1b,c that at *t* = 5 min, there is a sudden drop in resistivity *ρ_xx_* followed by a plateau with a corresponding rise in charge carrier density in BLG. This can be attributed to the AB‐to‐AA stacking transition of BLG and opening of the interlayer gap at the threshold intercalation density. This sudden rise in *n_e_* can be understood from our DFT calculations of Bader charge^[^ ^52^, ^53^ ^]^ on Eu atom and intercalation compound structure before and after this transition, see Materials and Methods, (Supporting Information) “Computation”. The Bader charge on Eu in AA stacked Eu‐graphene compound with equilibrium opening of 4.61Å is +1.3|e|, which is lower than +1.4 |e|, the charge in AB stacked Eu‐graphene compound with 3.68Å opening (the minimum interlayer gap where intercalation starts according to computational results, see Discussion Section) indicating another stage^[^ ^34^ ^]^ of Eu reduction. The charge carrier density keeps increasing until *t* ≈ 6 min when Eu ions stop forming further Eu‐intercalated compounds. Using Equation (1) and an assumption of constant rate of intercalation, we can also estimate the time required to complete the electrochemical intercalation, where *n_e_* = *n_Hall_* = 4.1 × 10^14^cm^−2^, is the Hall charge carrier density of intercalated BLG. We find *t* = 5.8 ± 0.8 min which is in good agreement with *t* = 6 min as observed from Figure 1c when *ρ_xx_* becomes constant and marks the completion of intercalation process.

## 2 Electrostatic Gate‐Controlled Reversible Eu Intercalation

**Figure 2:**
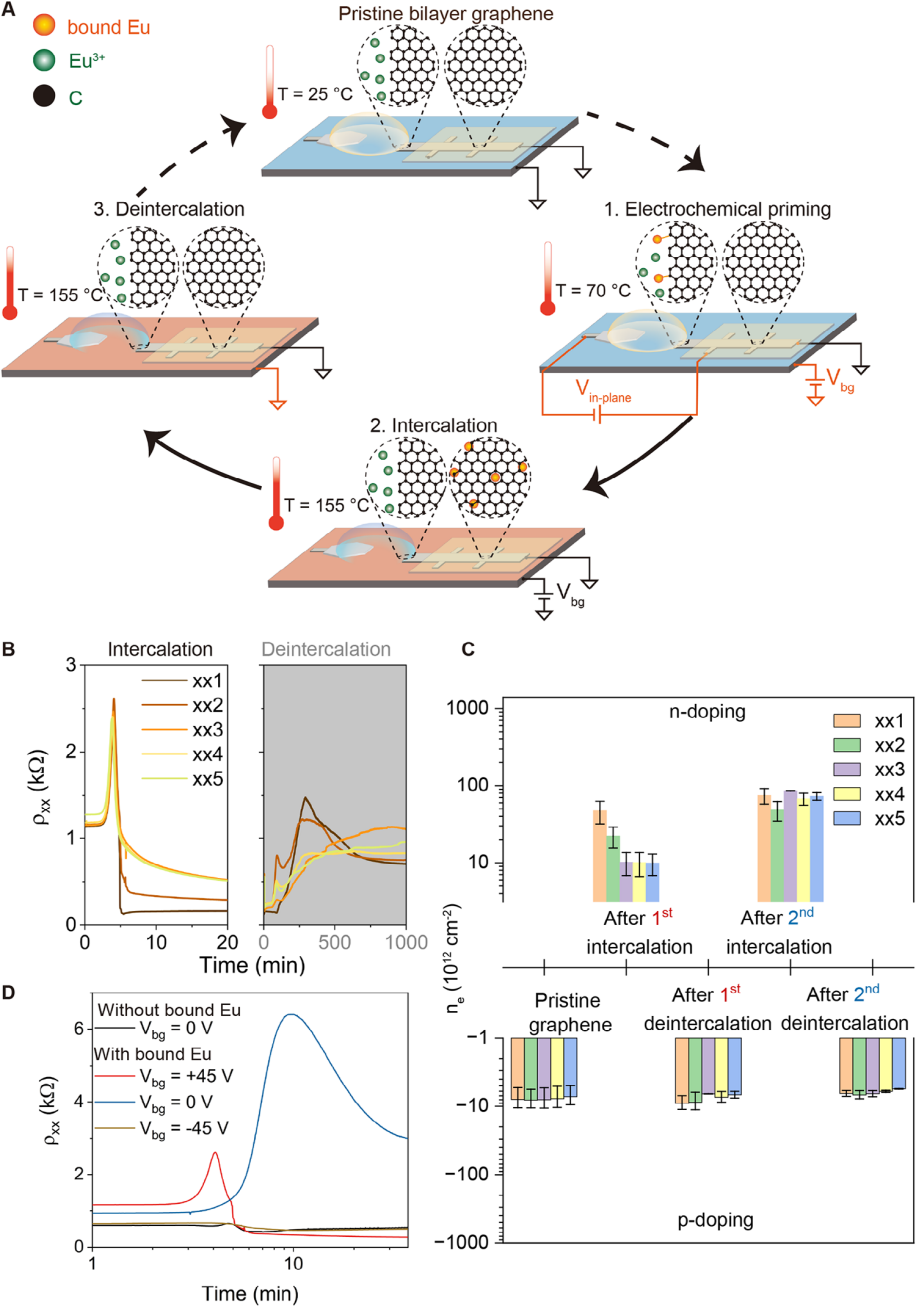
Gate controlled reversible intercalation of Eu into BLG. A) Schematics of the experiment. B) *ρ*_xx_(*t*) for steps 2 and 3 defined in panel a. During intercalation (step 2), *V*_bg_ = 45 V (BLG is negatively charged); during deintercalation (step 3), *V*_bg_ = 0 V. C) Charge carrier density with error margins of BLG device 2 before intercalation and after each intercalation and deintercalation steps. xxi (i = 1, 2, 3, 4) are neighbouring positions on the Hall bar‐shaped graphene with i = 1 as closest to the electrolyt0), *ρ*_xx_(*t*) for intercalation process (step 2 in panel a) of BLG at *V*_bg_ = 0 V where priming (step 1 in panel a) was skipped (black line), BLG at *V*_bg_ = 0 V (blue line), BLG at *V*_bg_ = 45 V (red line), and BLG at *V*_bg_ = ‐45 V (yellow line). Temperature profile: from 70 °C at *t* = 0 to 155 °C at *t* = 5.5 min.

The successful intercalation of Eu into BLG interlayer allows us to explore the intercalation process in more detail and to gain better control of the intercalation process, **Figure** **2**. Our devices benefit from a silicon back gate which can be used to electrostatically tune electronic transport properties of BLG independent of the electrochemical priming triggered by V_in‐plane_, to explore the intercalation‐deintercalation cycle. To this end, we experimentally divide the entire intercalation‐deintercalation cycle into three distinct steps (Figure 2a): electrochemical priming at low temperature (step 1), thermally activated gate‐controlled intercalation (step 2), and gate‐controlled deintercalation (step 3). For further details see Materials and Methods, (Supporting Information)“Electrochemical intercalation”. The evolution of *ρ_xx_* (*t*) along the device channel length for intercalation and deintercalations steps are shown in Figure 2b.

In step 1, in‐plane bias *V*_in‐plane_ = 8V is applied as before, but a lower temperature, *T* = 70 °C, is applied to prime the interface between BLG and electrolyte with Eu ions, since positively charged Eu^3+^ gets attracted to BLG and is partially reduced. At 70 °C, no intercalation is expected since the temperature is too low to initiate the intercalation. However, a considerable amount of Eu ions is reduced at the entrance of BLG, see Figure S3 (Supporting Information) and Materials and Methods, (Supporting Information) “Temperature control of Eu intercalation” for more details. The electrochemical priming step facilitates the formation of bound Eu at the entrance of vdW gap of BLG, which then intercalates into BLG interlayer by thermally activated diffusion process in step 2. We also found that the priming step 1 is critical. That is, if we skip step 1, then *ρ*_xx_(*t*) in step 2 would remain nearly constant throughout the intercalation process, indicating no intercalation has taken place, see black curve in Figure 2d.

In step 2, we remove *V*_in‐plane_ and ramp up the temperature to 155 °C, while applying back gate voltage *V*_bg_ +45, 0, or ‐45V. Here back gate voltage works as a control knob, in contrast to the solely temperature‐based electrochemical intercalation discussed earlier. We observe successful Eu intercalation featured by the peak in the *ρ_xx_* (*t*) (red curve in Figure 2d) and *I_bg_* (*t*) (Figure S4, Supporting Information) under *V_bg_* = 45V. No intercalation of Eu is observed under *V_bg_* = ‐45V when BLG is heavily p‐doped (yellow curve in Figure 2d). Even under *V_bg_* = 0V, noticeable changes in *ρ*_xx_(*t*) (blue curve in Figure 2d) featured by a delayed *ρ*_xx_(*t*) peak is observed. The resistivity at the end of intercalation (*t* ≈ 30 min) with *V_bg_* = 0V is higher than that the non‐intercalated BLG. This is likely due to the magnitude of the n‐doping being lower than initial p‐doping of BLG (assuming unchanged mobility), indicating partial intercalation under *V_bg_* = 0V.

In step 3, after successful intercalation at *V_bg_* = 45V, we initiate the deintercalation process by setting *V_bg_* = 0V. The intercalation and deintercalation processes are found to be highly reversible and repeatable, as shown in Figure S5a (Supporting Information). The charge carrier density n_e_ is plotted in Figure 2c, details are in Materials and Methods, (Supporting Information) “Electronic transport measurements.” We further observe in Figure 2c that during the first intercalation cycle, the highest charge carrier density reaches *n_e_* ≈ 4 × 10^13^cm^−2^. During the second intercalation cycle (also see Figure S5a, Supporting Information) the carrier density reaches *n_e_* ≈ 8.5 × 10^13^cm^−2^.

**Figure 3:**
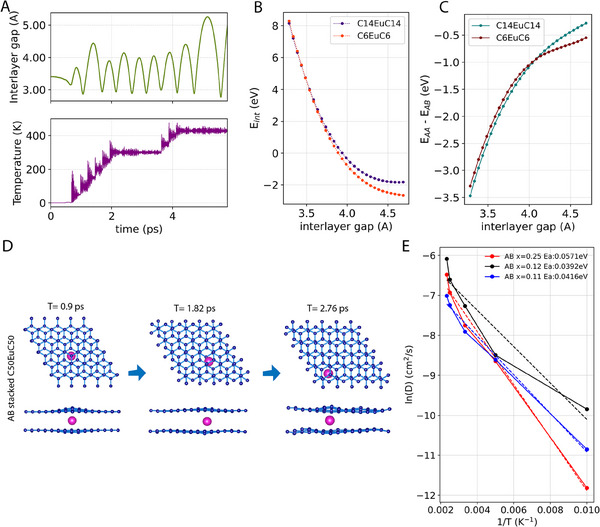
MD and DFT results for estimation of the dynamic interlayer gap, *E_in_*_t_, and stacking order during intercalation. A) (Bottom panel) Instantaneous temperature as a function of time during MD simulation using the Nose–hoover thermostat. Temperature is stabilized sequentially at room‐temperature (300 K) and 155 °C (428 K) for picoseconds. (Top panel) the vibration amplitude of breathing mode (average interlayer spacing) of the BLG as a function of time recorded during the temperature variation in the bottom panel. B). Intercalation energies for formation of C_14_EuC_14_ and C_6_EuC_6_ with varying graphene interlayer gaps. C). Difference in total energies between AA and AB stacking in C_14_EuC_14_ and C_6_EuC_6_ as a function of graphene interlayer gap. D) Trajectories of Eu atoms obtained from the molecular dynamic simulations on AB stacked C_50_EuC_50_. E) Arrhenius plot of diffusion coefficients calculated for different Eu densities (C_6_Eu_x_C_6_) in AB staked graphene.

Based on our observations of the reversible gate‐controlled intercalation, we propose the following mechanism for intercalation and deintercalation processes: (stage a): During priming step at 70 °C, Eu^3+^ ions at the entrance of BLG get reduced, forming bound Eu ions, but the interlayer gap is too narrow for Eu to intercalate (see Materials and Methods, (Supporting Information) “Temperature control of Eu intercalation”). The p‐doped nature of SU‐8 proximitised BLG with *n_e_* ≈ 10^13^ cm^−2^, as calculated from Figure S1c (Supporting Information), makes it harder for the Eu intercalation to happen. In‐plane priming voltage helps reducing the level of p‐doping, but still not sufficient to initiate intercalation. (stage b): As the temperature is raised, the interlayer gap increases substantially along with the kinetic energy of the intercalating Eu atoms. This makes intercalation of the bound Eu ions possible (see **Figure** **3**). With *V_bg_* = 45V, the BLG becomes much less p‐doped (even neutral or negatively charged), promoting the intercalation of bound Eu ions and diffusion of intercalated Eu ions into BLG until it reaches the percolation limit. With *V_bg_* = 0V, the much slower change in *ρ*_xx_(*t*) can be attributed to thermal activation. The p‐doped BLG (due to proximity to SU‐8) and the resulting low kinetic energy of intercalated Eu slows down the diffusion of Eu ions into BLG. At *V_bg_* = ‐45V, BLG is positively charged (heavily p‐doped), which builds up the energy barrier against intercalation of positive Eu ions. (stage c): With *V_bg_* = 0V, the device becomes p‐doped again. The thermal activation and doping‐driven kinetic energy together drive the deintercalation of Eu ions outside the BLG device. To support the proposed intercalation mechanism and deintercalation, we also carried out density functional theory (DFT) and molecular dynamics (MD) calculations, see Materials and Methods, (Supporting Information) “Computation.”

## 3 Discussion

To reconcile the experimental results and understand the role of temperature on the intercalation process, we consider a split‐step model using DFT and complementary Car‐Parrinello Molecular Dynamics (CPMD) calculations to describe the formation of Eu intercalation compound (see Materials and Methods, Supporting Information “Computation”): i) thermally activated expansion of interlayer separation in BLG, ii) intercalation of Eu into BLG interlayer with the energy *E_int_*, and iii) relaxation of Eu‐BLG system and interlayer gap expansion.

Figure 3a shows the interlayer gap between two graphene layers due to the breathing mode of thermal vibration under varying temperatures calculated using CPMD. At *t* = 0.45 ps, the equilibrium interlayer spacing (*c_eqb_*) is obtained by relaxing both the electronic and ionic degrees of freedom; with *c_eqb_* = 3.32Å our results closely mimic the experimental interlayer spacing. During the simulation, the temperature is first ramped to 300 K in steps with a mean interlayer gap of ≈3.5Å. When the temperature stabilizes at 428 K (155 °C), the elevated vibration amplitude of the bilayer increases the mean interlayer gap to 4Å allowing intercalation to commence. Figure 3b shows the intercalation energy $E_{int}=\left(E_{C_{N}Eu}-E_{Eu}-E_{C_{N}}\right)\;$ calculated for different interlayer gaps. Here, E_x_ denotes the total energy calculated using DFT for the compounds C_n_Eu, C_n,_ and isolated Eu atom. We calculate the intercalation energies for these compounds: C_14_EuC_14_ and C_6_EuC_6_ (see Materials and Methods, Supporting Information “Computation” for lattice structure selection). Formation of both C_14_EuC_14_ and C_6_EuC_6_ are thermodynamically favorable at interlayer gap above 3.68 Å where *E_int_* becomes negative. Moreover, for smaller gap openings, formation of C_14_EuC_14_ and C_6_EuC_6_ is almost equally likely, whereas for larger gap openings C_6_EuC_6_ formation is favorable. This explains why we get a mixture of these two compounds in the intercalated BLG. At this stage, both compounds are assumed to remain in the AαB stacking order.

We also observe the softening of bilayer graphene for easy intercalation after intercalation‐deintercalation cycles, previously observed by Astles et al.^[^ ^34^ ^]^ We tested a few cycles with deintercalation at V_bg_ = 0V as shown in Figure 2c. We find that residual resistivity after each intercalation‐deintercalation cycle gets lower as cycles progress, indicating more intercalation density with each cycle. To further investigate, we studied five intercalation‐deintercalation cycles under V_bg_ = +40V (intercalation) to ‐40V (deintercalation) (Please see Figure S5b, Supporting Information). We observe a reduction in resistivity for each cycle here as well. This softening happens most likely from residual Eu ions that keeps the interlayer gap large which helps intercalation in successive cycles.

Our DFT calculations show that the equilibrium interlayer gaps for C_14_EuC_14_ with AA and AB stackings are 4.33 and 4.38 Å, respectively; and for C_6_EuC_6_ these gaps are 4.61 and 4.74 Å, respectively. The calculated interlayer gaps are close to the interlayer space of Eu intercalated graphite in previous work, 4.87 Å (47, and references within). The differences in the total energies between AA and AB stacked configurations are plotted in Figure 3c. For both C_14_EuC_14_ and C_6_EuC_6,_ the AA stacking order is more favorable, leading to the spontaneous stacking order change. This also helps explain the structural change that is observed at *t* = 5 min, which brings the intercalated system to a relaxed position by increasing the interlayer gap and changing the stacking order from AB to AA. We have also studied the dynamics of the intercalated Eu atoms using ab‐initio CPMD with both AA and AB stacked BLG. We find that the diffusion of Eu ions in AA‐stacked BLG is much slower compared to that in AB‐stacked BLG. Trajectories and formation of bubbles in AB stacked BLG is shown in the snapshots in Figure 3D. In‐plane diffusion coefficients of Eu atoms are calculated at temperatures 100, 200, 300, 400, and 428 K (155 °C) in AB BLG as time derivative of Mean Square Displacement (MSD) following earlier intercalation studies.^[^ ^54^ ^]^ We find that the diffusion coefficients in AB BLG increase almost exponentially with the temperature, which leads us to check if Eu intercalation follows Arrhenius behavior. The Arrhenius plot in Figure 3E shows that the activation barrier (E_a_) increases with increasing density of Eu intercalation.

To understand the driving mechanism behind the reversible intercalation/deintercalation, we consider the correction to intercalation energy ($E_{int}^{corr}$) by doping from back gate:^[^ ^34^ ^]^

(2) $$E_{int}^{corr}=E_{int}\left(T\right)+q\Delta \mu$$

where intercalation energy (*E_int_*) is a dynamic term and a function of temperature *T*, *q* is the charge at the BLG due to doping, and *Δμ* denotes the change in the chemical potential of the electrons transferred. The energy associated with this charge transfer can be approximated by the energy stored in the dielectric (assuming the contribution from the quantum capacitance of the BLG layer to be much smaller than that of the SiO_2_ layer). Therefore, we can write $q\Delta \mu \approx -\mathrm{sgn}\left(V_{bg}\right)\;\frac{1}{2}CV_{BG}^{2}$ where *V_BG_* is the back gate voltage and areal capacitance of SiO_2_ $C=\frac{\varepsilon \varepsilon_{r}}{t}$, in which *t* = 290 nm is the thickness of the dielectric layer, and *ɛɛ_r_* gives the dielectric constant for SiO_2_. For our device *C* = 1.19 × 10^−4^F m^−2^ and corresponding charging energy is 0.0075 eV Å^−2^ for *V_bg_* = 45 V. For C_14_EuC_14_ unit cell with an area 21.46 Å^2^, varying the back gate voltage within ±45V changes the Gibb's free energy by ± 0.161 eV and for C_6_EuC_6_ the Gibbs free changes by ± 0.069 eV, with a positive back gate voltage making *E_int_* more negative. This energy is much larger than the thermal energy of the Eu ions themselves which can be approximated in 2D by $E_{th}=2\cdot \frac{1}{2}k_{b}T\approx \;0.025$ eV at 300 K. Therefore, for both intercalation and deintercalation processes, the back gate voltage alters the intercalation energy *E_int_*. The term *E_int_* also keep on changing at elevated temperatures as the breathing mode changes the interlayer spacing (see Figure 3a,b). The temperature results in the total energy of the system fluctuating around zero, and the gate voltage correction governs whether intercalation or deintercalation will take place. Therefore, for intercalation and deintercalation, an elevated temperature is necessary, and that is why Eu does not deintercalate spontaneously at room‐temperature.

Our findings open several promising avenues for future exploration. Because devices operate at temperatures higher than those used in conventional electrochemical tests, a quantitative assessment of the electrolyte and the SU‐8 protective layer durability will be an important next step. Evaluating performance over thousands of cycles will likewise clarify long‐term stability. Expanding the architecture – both by adding more layers and by increasing the device's overall size – offers another exciting direction. Finally, assessing the potential for applications could be done by systematically examining device efficiency, cost (given the rare‐earth elements involved), reliability, and robustness.

In summary, we have developed a reversible europium intercalation method for BLG that utilises both electrostatic and thermal control. This approach enables precise and controllable Eu intercalation and deintercalation between van der Waals gaps at elevated temperature, expanding the potential applications for 2D materials beyond conventional intercalants and conditions. Our findings also pointed out the temperature effort on reducing insertion energy via expanding interlayer space and the ionic state of intercalated metal. Our findings reveal insight into temperature and voltage on electrochemical intercalation and paving the way for advanced graphene‐based heterostructures with potential applications in next‐generation electronics and quantum technologies.

## Conflict of Interest

The authors declare no conflict of interest.

## Author Contributions

M.F. and A.M. conceived the study. M.F. and Q.D. developed the methodology. M.F., A.B., C.M., and Y.S. carried out the investigation. M.F. and A.B. were responsible for visualization. Formal analysis was conducted by M.F., A.B., and A.S. Funding was acquired by A.M. and Q.Y. A.M. and Q.Y. administered the project. Supervision was provided by A.M., Q.Y., and K.S.N. The original draft was written by M.F., A.M., A.B., Q.Y., A.R., K.S.N., and Q.D. All authors—M.F., Q.D., A.B., C.M., A.S., Y.F., I.T., Y.S., A.R., K.S.N., Q.Y., and A.M.—contributed to reviewing and editing the manuscript. Data Availability Statement

## Supporting information



Supporting Information

## Data Availability

The data that support the findings of this study are available from the corresponding author upon reasonable request.
