# Supplementary material for: Rare‐Earth Ion Intercalation in Graphene via Thermal and Electrostatic Control
Source: Adv Mater. 2025 Jul 6;37(38):2502417. doi: 10.1002/adma.202502417 (PMC12464637; doi:10.1002/adma.202502417)
Supplement: Supplementary file 1 — Supporting Information [file ADMA-37-2502417-s001.docx]

Supplementary Materials for

**Rare-earth ion intercalation in graphene via thermal and electrostatic control**

Mengjie Feng^1,2^*, Qing Dai^1,2^, Anupam Bhattacharya^1^*, Ciaran Mullan^1^, Amit Singh^1^, Yangming Fu^1,2^, Ivan Timokhin^1,2^, Yanmeng Shi^1,3,4^, Alexander Rudnev^5^, Kostya S. Novoselov^1,2,6^*, Qian Yang^1,2^, Artem Mishchenko^1,2^*

^1^Department of Physics and Astronomy, University of Manchester, Manchester M13 9PL, UK

^2^National Graphene Institute, University of Manchester, Manchester M13 9PL, UK

^3^State Key Laboratory of Semiconductor Physics and Chip Technologies, Institute of Semiconductors, Chinese Academy of Sciences, Beijing 100083, China

^4^Center of Materials Science and Optoelectronics Engineering, University of Chinese Academy of Sciences, Beijing 100049, China

^5^Department of Chemistry, Biochemistry and Pharmaceutical Sciences, University of Bern, 3012 Bern, Switzerland

^6^Institute for Functional Intelligent Materials, National University of Singapore, Singapore, 117544, Singapore

*Corresponding authors; emails: fengmj@buaa.edu.cn, anupam.bhattacharya@manchester.ac.uk, kostya@nus.edu.sg, artem.mishchenko@manchester.ac.uk

Materials and Methods

**Feasibility of electrochemical intercalation of lanthanides into bilayer graphene**

Several factors, including size effect, electronegativity, and standard electrode potential, are known to influence the feasibility of electrochemical intercalation. **fig. S6** shows that rare earth metals have comparable standard electrode potential (*55,56*), atomic and ionic radii (*57,58*), and electronegativity (*59*) to those of alkali and alkaline earth metals. Experimental studies have repeatedly demonstrated the successful electrochemical intercalation of alkali metal ions (*60,61*) and alkali earth metal ions (*62,63*) into layered materials. However, the electrochemical intercalation of multivalent ions (*64*) is often hindered by the formation of solid electrolyte interface layer during the intercalation process. Moreover, compared to alkali metals, rare earth metals have lower electronegativity and stronger electrostatic interactions with the host lattice (*65*). Consequently, while the intercalation of rare earth metal ions is feasible, it is more challenging than the electrochemical intercalation of alkali metal ions. In this work we report electrochemical intercalation of Eu ions inside bilayer graphene.

**Device fabrication**

The intercalation device is a lithium-ion battery-like cell mounted on p-doped silicon substrate with a 290 nm thick SiO_2_ layer; schematics and micrographs of one of our devices are shown in **Fig. 1a**. The device mainly consists of electrolyte, a Hall bar-shaped bilayer graphene working electrode, and a thick graphite flake counter electrode. Monolayer (MLG) (use of MLG device is described in ‘**Analysis of intercalation pathway’**) and bilayer graphene (BLG) are mechanically exfoliated from bulk graphite (NGS Naturgraphit GmbH) using adhesive tape (Scotch Magic tape, 3M) onto silicon substrate as described in (*28)*. Both Raman spectroscopy (*66)* and optical contrast (*67)* are used to differentiate the MLG and BLG (see **Methods, Raman measurement**). The Hall bar geometry of the bilayer graphene and metal contacts are fabricated via a standard graphene patterning process, which includes polymethylmethacrylate (PMMA) mask patterning by e-beam lithography, O_2_-plasma etching, Cr/Au (3 nm/50 nm) metal contact deposition, and lift-off. The graphite counter electrode, with an accessible area larger than 200 μm^2^, is located microns away from the working electrode (Fig. 1a). An electrochemically stable SU-8 film partially covers the bilayer graphene and metal contacts to isolate them from the electrolyte. The electrolyte is drop casted on the substrate, specifically in the SU-8 window, connecting the counter electrode and the bilayer graphene. To distinguish the intercalation component, we prepared two polyethylene oxide (PEO)-based electrolytes: (1) an electrolyte (*68)* consists of europium triflate (98%, Sigma-Aldrich) and PEO (average molecular weight ≈900,000, Sigma-Aldrich) *w/w* = 1:2.2, (2) pure PEO electrolyte without Eu.

**Electronic transport measurements**

The electronic transport measurements of Hall-bar shaped graphene consist of three parts: (1). Gate voltage dependence of longitudinal resistivity, *ρ*_xx_(*V_g_*), at room temperature. (2). In situ time dependence of longitudinal resistivity *ρ*_xx_(*t*), monitored during the intercalation and deintercalation process. (3). Magneto transport measurements at *T* = 0.3 K. All measurements were conducted in a high vacuum environment (< 10^-6^ mbar) using a standard lock-in technique with 100 nA excitation current at 17.27 Hz, employing Stanford Research SR830 lock-in amplifiers. A 1 MΩ resistor is connected between lock-in amplifier and BLG to output the typical root mean square amplitudes *I* = 100 nA. The longitudinal resistivity, *ρ*_xx_, is calculated using the equation $\rho_{xx}=\frac{WU_{xx}}{LI}$, where *W* is the width of the bilayer graphene device, *L* is the length between measurement contact pairs, *U*_xx_ is the longitudinal voltage drop, and *I* is the excitation current. *ρ*_xxi_ is measured at several different neighbouring positions *i*, with *i* = 1 being the closest to the electrolyte.

The intrinsic charge carrier density, *n_e_*, and electron mobility, *μ*, were obtained from fitting the equation $\rho_{xx} =\frac{1}{\sigma_{xx}} = \frac{1}{e(n_{e}+n_{bg})\mu}=\frac{1}{en_{e}\mu+ \mu CV_{bg}}$, where *σ*_xx_ is the longitudinal conductivity, *n_bg_* is charge carrier density corresponding to gate voltage *V*_bg_, *e* is the electron charge, and *C* is the capacitance of the back gate dielectric. For 290-nm-thick SiO_2_ back-gate dielectric, the capacitance *C* is 1.2×10^-8^ F cm^-2^. The gate voltage sweep rate is 5 V/min unless otherwise specified. Charge carrier density *n_e_* for **Fig. 1c** is estimated from **fig. S1b** using the above equation at room temperature. Then, the device is heated to 155 C, and the mobility is estimated from *μ_0_* = 1/*ρ_xx_en_e_* where *ρ_xx_* is the resistivity of device at 155 C.

Magnetotransport measurements at cryogenic temperatures were performed with a He^3^ cryostat by Oxford Instruments with a perpendicular magnetic field, *B*, up to 18 T. In cryogenic measurements, the transverse resistivity, *R_xy_*, is also measured to extract the charge carrier density *n_Hall_* = *BI*/*U_xy_e* = *B*/*R_xy_e*, where *U*_xy_ is the transverse volage drop, and the back gate and *B* field dependence of longitudinal resistivity, *ρ_xx_*, is measured and plotted. From the frequency *B_F_* of Shubnikov-de Haas oscillations of *ρ_xx_* as a function of *B^-1^*, corresponding charge carrier density is extracted from *n*_SdH_ = $\frac{geB_{F}}{h}$, where *g* = 4 is the Landau Level degeneracy, *e* is the elementary charge, and *h* is the Plack’s constant.

**Raman measurements**

Raman spectroscopic analysis is performed using a micro-Raman spectrometer (Horiba XploRA^TM^ PLUS) with an unpolarised laser beam of wavelength *λ* = 532 nm. The laser power is kept below 1 mW to minimise the sample damage (*69*). The laser beam is focused onto the sample’s surface through a ×100 objective, resulting in a spot size less than 1 µm. Raman mapping is carried out with a step size of 1 µm. The obtained spectral data were processed using Labspec software, which includes baseline subtraction and fitting with Gauss-Lorentz function.

The presence of graphene quenches photoluminescence signals (*70)*, and the Pauli blocking effect in metal-filled few-layer graphene (*71)* reduces photon adsorption and affects the Raman peaks (*72)*. To ensure clear signal changes in the Raman spectra, both the intercalation compound and few-layer graphene were exposed to air for more than 12 h prior to measurements. In the spectra of the oxidized intercalation compound, the D peak associated with defects in graphene is observed. Raman spectra of few-layer graphene remained unchanged after exposure to air due to its stability. The use of Raman spectroscopy of intercalation compound is described in ‘**Analysis of intercalation pathway**’.

**Electrochemical intercalation**

Prior to intercalation, the device is stored in a high vacuum environment (< 10^-6^ mbar) for more than 3 hours to remove surface water and oxygen. During the intercalation process, the cell is placed on a hot plate in the high vacuum environment. We introduce two different procedures for Eu intercalation, as described below.

The first is a one-step intercalation - Eu electrochemical intercalation at high temperature, where the device is directly heated to the target temperature of intercalation experiments. A voltage *V_in-plane_* = 8 V is then applied between the counter electrode and bilayer graphene contacts closest to the electrolyte at *t* = 0.1 min. The voltage is controlled by a Keithley SourceMeter 2614a.

The second procedure is the temperature-activated gate-controlled reversible Eu intercalation and deintercalation. This is performed following the steps as depicted in **Fig. 2a**:

1. Electrochemical priming at lower temperature: a voltage *V*_in-plane_ = 8 V is applied between counter electrode and Hall-bar shaped graphene device at 70 °C for 1 h.
2. Intercalation activated by temperature and controlled by gate voltage: *V_in-plane_* is unplugged, the gate voltage is swept to the target voltage: +45 V, 0 V or -45 V, at room temperature (25 °C) followed by temperature rising to 155 °C. The temperature is maintained until the end of the intercalation experiment.
3. Deintercalation: The step 2 is repeated, except the gate voltage is set to 0 V and the temperature rise to 155 °C.

In the second procedure, the temperature rise starts from *t* = 0.1 min, unless stated otherwise.

**Temperature control of Eu intercalation**

To determine the optimal temperature for electrochemical intercalation, experiments were also performed at various temperatures at *T* = 70, 80, 130, 140, 155, and 185°C. Intercalation process at different temperatures were performed with BLG devices (device 2, device 12 and devices 5 - 7). The electronic transport properties and Raman spectra of BLG before and after intercalation are shown in **fig. S3**. In the plots of *ρ*_xx_(*t*), *V_in-plane_* is applied from *t* = 0.1 min.

During the intercalation process at 70°C, *ρ*_xx_(*t*) remained constant (**fig. S3a**). The charge carrier densities extracted from the gate voltage dependence of *ρ_xx_*(*V*_bg_) before and after intercalation were similar (**fig. S3b**). The Raman spectra of MLG and BLG after intercalation at 80 °C perfectly overlap with their spectra before intercalation (**fig. S3c and d**), indicating no intercalation has taken place. When the intercalation temperature is increased to 130°C, *σ_xx_* still did not show significant difference for an extended period (**fig. S7a**). While at 140°C, *σ_xx_*(*t*) increased slowly with time, indicating the onset of Eu intercalation (**fig. S7b**). The slow intercalation rate may be attributed to the slow diffusion at this temperature. At *T* = 185°C, the *σ_xx_*(*t*) gradually increased with time to around 4 mS revealing the Eu intercalation with time (**fig. S7c**). However, this temperature is close to the decomposition temperature of PEO (*48,73*), so the Eu intercalation fades as the PEO decomposes. These observations are consistent with the DFT and molecular dynamic calculations presented in **Fig. 3**, showing the temperature effects on Eu intercalation.

**Eu intercalation voltage**

To determine the Eu intercalation voltage, the electrochemical ‘intercalation’ experiments of bilayer graphene (device 4) is also performed at lower in-plane bias: 1.8 V and 3 V in addition to 8V. Compared with the Raman spectrum of pristine BLG, the Raman spectra of BLG after ‘intercalation’ process at both 1.8 V and 3 V do not show a significant difference (**fig. S8a**) . The in-plane current flow between BLG and counter electrode decreases with time during the ‘intercalation’ process at 1.8 V or 3 V, but it’s comparably stable at 8 V and higher than that at 1.8 V or 3 V after a long term ‘intercalation’ process (**fig. S8b**). That may be because the formation of solid electrolyte interface at a lower in-plane bias blocks the current flow and the intercalation pathway (*74*), while a higher in-plane bias enables the pathway. Overall, a higher voltage (8 V) is required for Eu intercalation**.**

**Analysis of intercalation pathway**

During the intercalation of bilayer graphene, Eu has three potential intercalation pathways: the space between top graphene and SU-8 protective layer, BLG interlayer (vdW gallery), and the space between bottom graphene layer and the SiO_2_ layer of Si substrate. To estimate the contribution of each pathway, we performed a range of additional experiments, including those with MLG, where the vdW gallery is absent. To this end, monolayer and bilayer graphene devices were subjected to the intercalation procedure at 155°C, and their optical and electronic transport properties were compared and analysed, as shown in **Fig. 1** and **fig. S2**. During this process, *ρ*_xx_(*t*) of MLG device 11 shows an order of magnitude smaller changes as compared with BLG devices 3 and 1, cf. **fig. S2a,** and **Fig. 1b**. The difference between charge carrier densities before and after the intercalation of MLG device, extracted from *ρ*_xx_(*V*) (**fig. S2b**), is 1.3 × 10^13^ cm^-2^ transitioned from p-doped with n = 1.5× 10^12^ cm^-2^, to n-doped 1.17 × 10^13^ cm^-2^, which is also an order of magnitude smaller than that in the BLG case.

The Raman spectrum of the oxidized BLG intercalation compound (device 4) showed a notable D peak, which is absent in MLG region of the same device, **fig. S2e**. We performed intercalation of another device consisting of BLG and MLG region. The Raman maps of D peak intensity and the intensity ratio of D to G peaks measured after device oxidation in air are shown in **fig. S2f**. Both maps show a clear difference between BLG and MLG, further confirming the absence of intercalation in the case of MLG.

The charge carrier density of intercalated BLG (device 3) extracted from the Hall effect (**fig. S2c**) is *n_1_* = *n_Hall_* = 4×10^14^ cm^-2^ (see **Methods, ‘Electronic transport measurements’**). In device 3 we observed two sets of oscillations of *ρ_xx_*(*B*), both periodic in 1/*B*, confirming them as Shubnikov-de Haas (SdH) oscillations, plotted in **fig. S2g**, together with two corresponding sets of Landau fans. By analysing the charge carrier density origins and spacings of these two sets of Landau fans, we extracted their corresponding carrier densities, *n_2_* = -7.8 × 10^12^ cm^-2^ (Landau levels marked with dark lines) and *n_3_* = n_SdH_ = 2.6 × 10^13^ cm^-2^ (Landau levels marked with golden dash lines). Notably, *n_2_* is close to *n* = -5.5 × 10^12^ cm^-2^ for that of BLG (device 3) before intercalation, which corresponds to the SU-8 doping. And *n*_3_ is similar to the charge carrier density change of intercalated MLG with *n_e_* = 1.4 × 10^13^ cm^-2^ corresponding to intercalated Eu doping from the graphene-silicon substrate interface.

Assuming the presence of three distinct types of charge carriers in the BLG intercalation compound, we fitted the magnetic field dependence of conductivity with a multicarrier model (*75*):

$\sigma_{xx}=\sum_{i=1}^{3} \frac{n_{i}q\mu_{i}}{1+\mu_{i}^{2}\boldsymbol{B}^{2}}$ (2)

$\sigma_{xy}=\sum_{i=1}^{3} \frac{n_{i}q\mu_{i}^{2}\boldsymbol{B}}{1+\mu_{i}^{2}\boldsymbol{B}^{2}}$ (3)

Here, σ_xx_ and σ_xy_ are longitudinal and transverse electrical conductivities, respectively. *μ_i_* is the charge carrier mobility of *i*^th^ (1 ≤ i ≤ 3) type carrier, *n_i_* is the density of each of the three types of charge carriers. *n*_1_, *n*_2_ and *n*_3_ were input into the fitting model, and *μ_1_*, *μ_2_* and *μ_3_* are fitting parameters. The experimental results were fitted well with the multicarrier model, as shown in **fig. S2d**.

Hence, from this analysis we can further confirm that Eu predominantly intercalates in the BLG interlayer and that the intercalation into graphene-silicon substrate or graphene-SU-8 polymer interfaces are negligible.

**Computation**

DFT calculations were performed using Quantum Espresso package (*76*) based upon the plane-wave pseudopotential method with PAW pseudopotentials. The exchange-correlation potentials were modelled using Perdew–Burke–Ernzerhof (PBE) functional. The pseudopotential is generated with a scalar relativistic approximation. Structural relaxation is carried out until the convergence thresholds for energy and force were reached at 10^-5^ Ry and 10^-4^ Ry/Bohr, respectively. During all calculations, kinetic energy cutoff for wavefunction is kept at 50 Ry and same for charge density and potential is 350 Ry. Dispersion correction for van-der Waals interaction is modelled using nonlocal vdw-df functional optB88 by Klimes et al (*77*). Several other dispersion corrections e.g. DFT-d3, DFT-d2 and vdw-df2 were tested but optB88 is chosen because of its accurate prediction of 3.32 Å as interlayer gap in Bernal graphene. Monkhorst-Pack k-mesh $13\times13\times1$ is used for simulation of the BLG graphene with 4 atoms, and $7\times7\times1$ is used for calculations with C_14_EuC_14_ and C_6_EuC_6_ supercells. The intercalation energy *E_i_* is defined as $E_{int}=\left( E_{C_{N}Eu}-E_{Eu}-E_{C_{N}} \right)$, where $E_{C_{N}Eu}, E_{Eu}, E_{C_{N}}$are the total energies of intercalation compound in AαB stacking order containing N carbon atoms and a single Eu atom, one single Eu atom and BLG containing N carbon atoms.

The thermally activated interlayer gap opening is studied with Car-Parrinello molecular dynamics (CPMD) implemented in the Quantum Espresso package. A 64-atom supercell of AB stacked BLG is used for the simulation. Initially, a microcanonical ensemble is simulated in multiple steps (electronic relaxation, multistage ionic relaxation) to gain thermal stability. A convergence criterion for force of $3\times{10}^{-4}$Ry/bohr is kept for achieving the thermal equilibrium. A Nose-Hoover thermostat is used to numerically bring the system temperature to first 300K (room temperature) and then to 428 K (155°C) in multiple steps of 50 K. The canonical ensemble is assumed for all elevated temperatures.

Bader charge analysis (*52,53*) is employed to calculate the charge population of intercalated Eu in BLG. The analysis is performed by running the Bader charge analysis code (*42*) following the Quantum Espresso package. The lattice structure of intercalation compound, in which Eu atom is positioned at the hexagon hole site of AA-stacking BLG interlayer and at the hexagon hole site of bottom layer graphene in AB stacking BLG interlayer, is used for the calculations. All the structures were relaxed to their minimum energy configurations.

We carried out DFT calculations on two Eu intercalation compounds: 1. $\left( \sqrt{3} \times\sqrt{3} \right)R30^{\circ}$ C_6_EuC_6_, and 2. $\left( \sqrt{7} \times\sqrt{7} \right)R19.1^{\circ}$ C_14_EuC_14_. This selection is based on the following reasons. Eu forms C6Eu when intercalated in graphite (*46,47*). Previous studies of Li electrochemical intercalation have shown formation of C_6_LiC_6_ and C_14_LiC_14_ compounds (*29,34*). The intercalation energies of C_6_EuC_6_ and C_14_EuC_14_ are comparable as shown in **Fig. 3b** when interlayer gap is small.


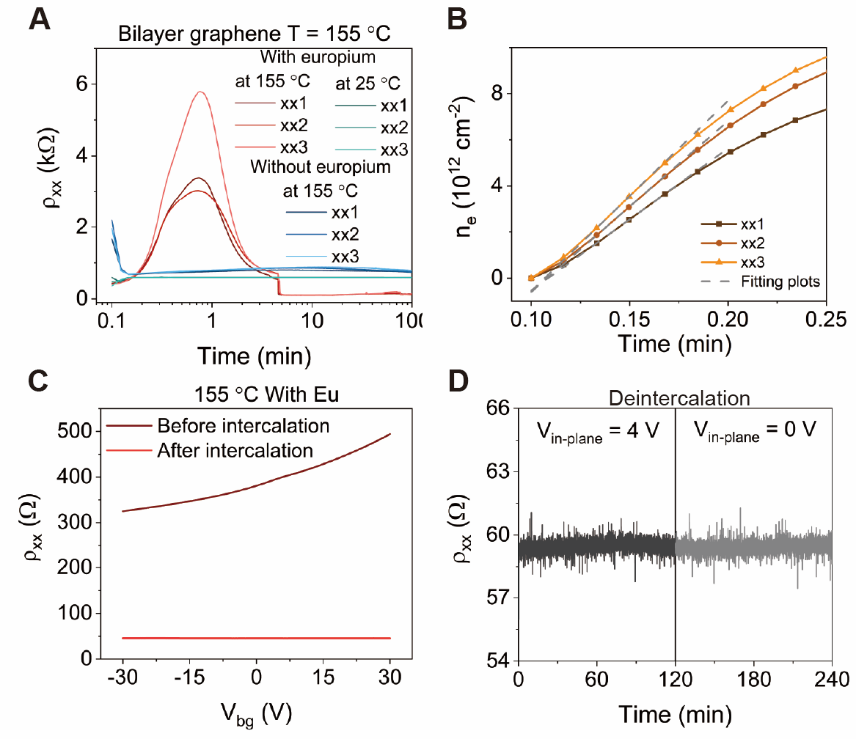


**fig. S1: Europium intercalation and deintercalation at 155 °C probed by electronic transport measurements**. **A,** Time dependence of longitudinal resistivity of bilayer graphene intercalated with europium at 155 °C (red lines, device 1), without europium at 155 °C (blue lines, device 8) and with europium at 25 °C (purple lines, device 2). **B,** Time dependence of n_e_(t) in the range of 0.1 min < t < 0.25 min and linear fitting plots. **D,** Gate voltage dependence of longitudinal resistivity of BLG before and after intercalation with europium at 155 °C. **D,** Time dependence of longitudinal resistivity of intercalation compound during deintercalation motivated by reducing intercalation voltage to 4 V or 0 V (device 9).


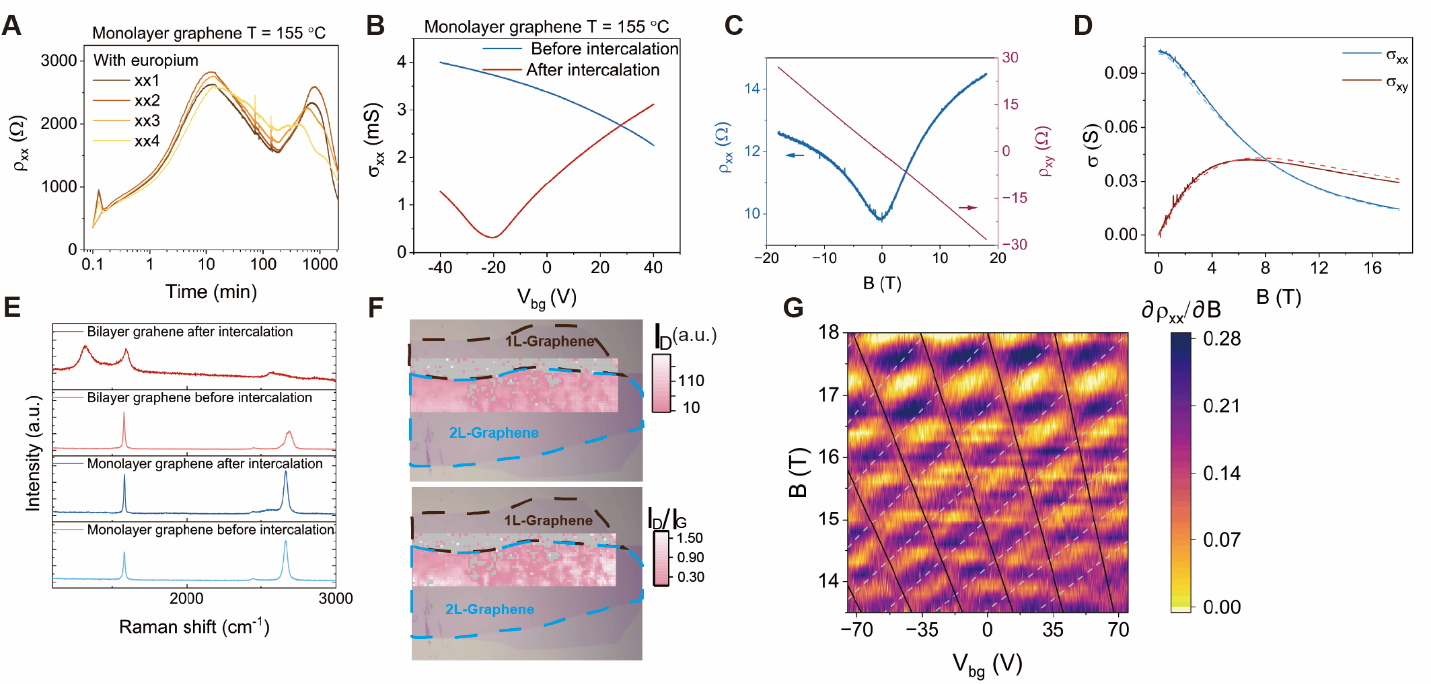


**fig. S2: Analysis of europium intercalation pathways.** **A**, Time dependence of longitudinal resistivity of europium ‘intercalated’ MLG device 11 at T = 155 °C. **B**, Gate voltage dependence of conductivity of MLG device 11 before and after europium ‘intercalation’ at T = 155 °C. **C**, Magnetic field dependence of longitudinal resistivity and Hall resistivity of europium intercalated BLG device 3 at T = 155 °C. **D**, Magnetic field dependence of conductivity of europium intercalated BLG device 3 at T = 155 °C and its fitting plots with two charge carrier model. **E**, Raman spectra of oxidized Eu ‘intercalated’ MLG and BLG and pristine MLG and BLG (device 4) **F**, Microscopy image and Raman map of D peak intensity and the intensity ratio of D peak and G peak, I_D_/I_G_, of selected device with europium ‘intercalated’ MLG region and BLG region excited by laser with wavelength λ = 532 nm. **G**, Landau fan diagram of BLG 𝜕ρ_xx_/𝜕B(*V*_bg_,*B*) after intercalation. The magneto resistivity measurements of Eu intercalated BLG device 3 was performed at *T* = 0.3 K, and both back-gate voltage dependence of longitudinal resistivity measurements and the Raman spectroscopy measurements were performed at room temperature.


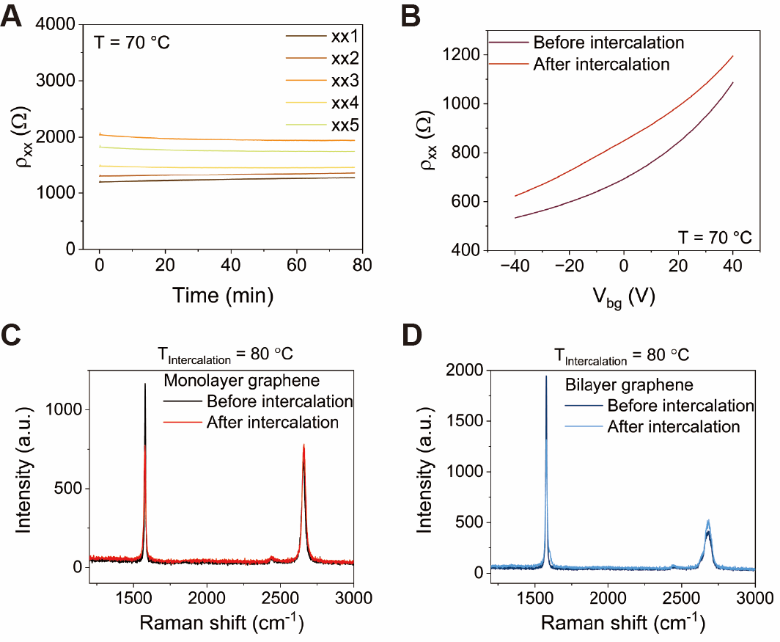


**fig. S3:** **Electrochemical intercalation at lower temperatures (70 °C and 80 °C).** **A,** Time dependence of longitudinal resistivity of bilayer graphene device 2 intercalated at 70 °C started from t = 0 min. **B,** Gate voltage dependence of longitudinal resistivity of bilayer graphene before and after the intercalation at 70 °C for 75 min. **C,** Raman spectra of MLG (device 12) before and after intercalation at 80 °C for 12 h. **D,** Raman spectrums of bilayer graphene (device 12) before and after intercalation at 80 °C for 12 h.


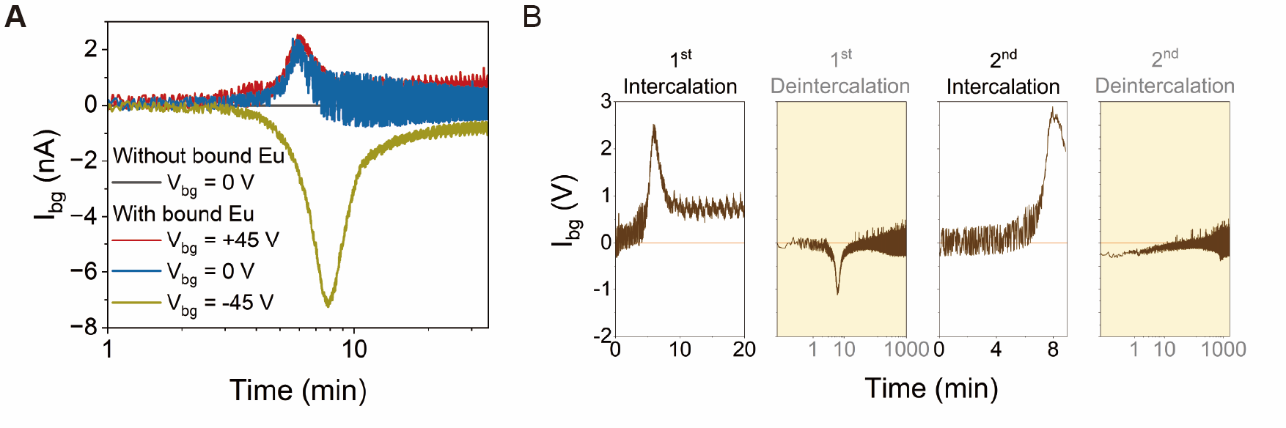


**fig. S4**: **Electrochemical reaction of split-step intercalation probed by time dependence of back gate current (device 2).** **A,** I_bg_(t) for intercalation process (step 2 in panel a) of BLG at V_bg_ = 0 where priming (step 1 in panel a) was skipped (black line), V_bg_ = 0 (blue line), V_bg_ = 45 V (red line) and V_bg_ = -45 V (yellow line). Temperature profile: from 70 °C at t = 0 to 155 °C at t = 5.5 min. **B,** I_bg_(t) for steps 2 and 3 during intercalation (step 2), V_bg_ = 45 V (BLG is negatively charged); during deintercalation (step 3), V_bg_ = 0 V.


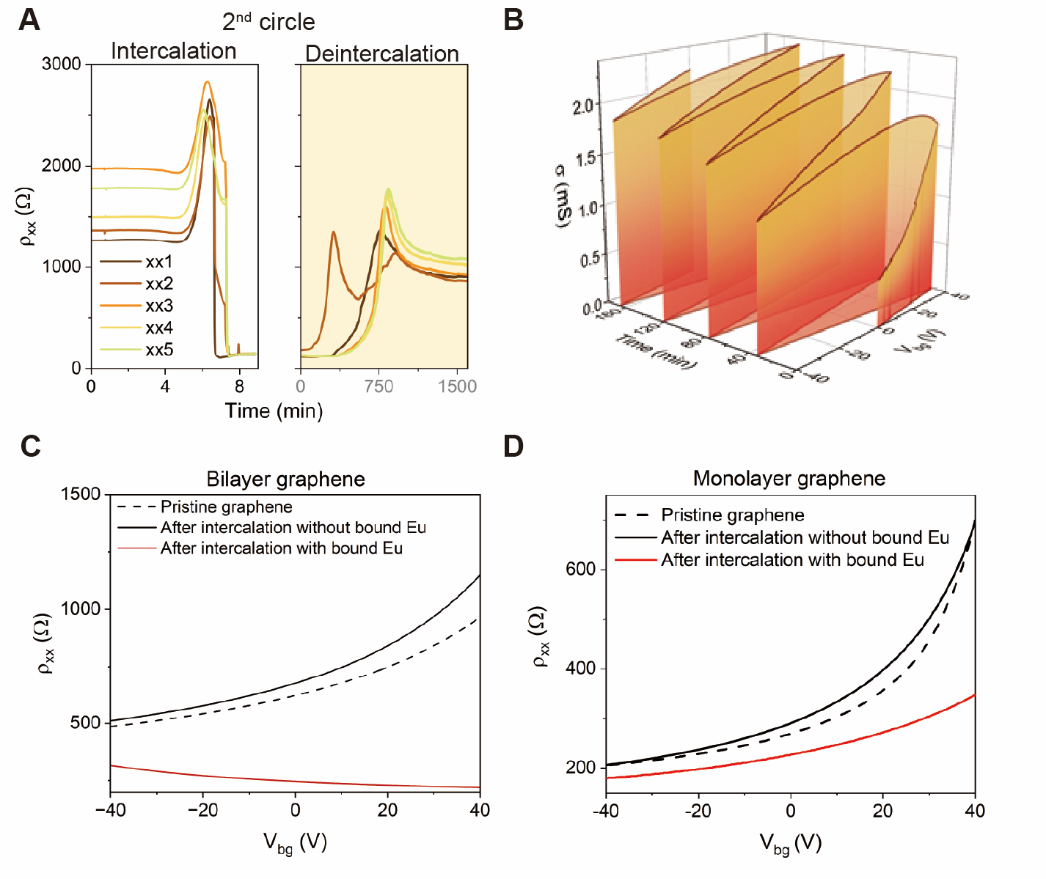


**fig. S5: Thermally activated and gate controlled reversible europium intercalation and deintercalation.** **A,** Time dependence of longitudinal resistivity of bilayer graphene device 2 revealing the 2^nd^ intercalation and deintercalation circle. In intercalation is triggered by V_bg_ = 45 V, and the deintercalation was controlled by V_bg_ = 0 V. This is the second cycle of the intercalation and deintercalation of the same device as in Fig. 2b in the main text. **B,** Gate voltage and time dependence of longitudinal resistivity of intercalated bilayer graphene device 2 at room temperature. The bilayer graphene was pre-intercalated at 155 °C to expand their interlayer space, but its interlayer space was not completely filled with europium before this measurement. **C,** Back-gate voltage dependence of longitudinal resistivity of pristine graphene device 2 (black dash), bilayer graphene after electrochemical intercalation but skipping electrochemical priming step (black line) and bilayer graphene after split-step electrochemical intercalation (red line). **D,** Back-gate voltage dependence of longitudinal resistivity of pristine MLG device 10 (black dash), MLG after electrochemical intercalation but skipping electrochemical priming step (black line) and BLG after split-step electrochemical intercalation (red line).


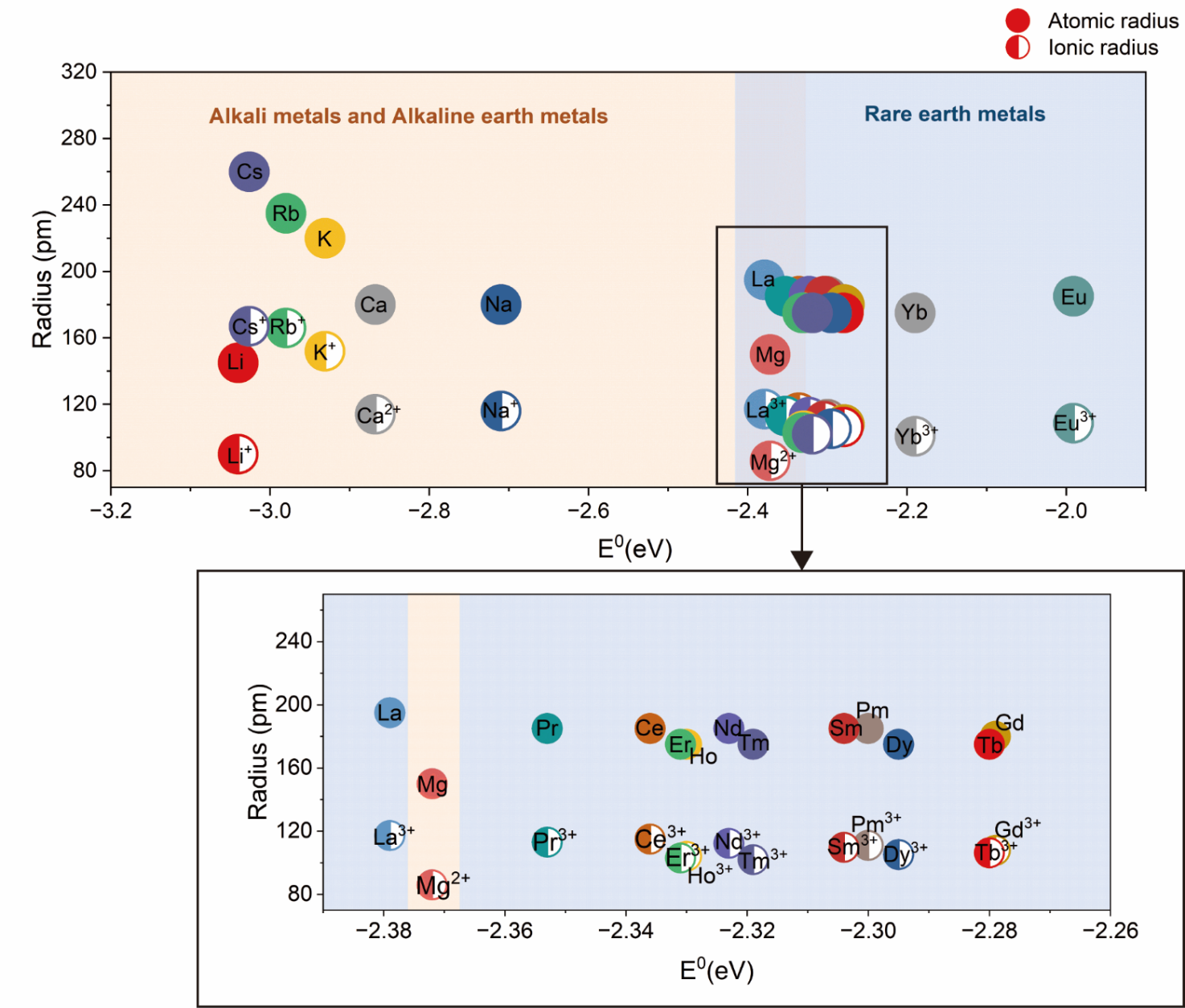


**fig. S6:** **Analysis of the feasibility of electrochemical intercalation of rare earth metals.** Standard electrode potentials relative to standard hydrogen electrode, atomic and ionic radii of alkali metals (Li, Na, K, Rb, Cs), alkali earth metals (Ca and Mg) and rare earth metals. The standard electrode potential E^0^ is the reduction potential of X^3+^ to X, and X is the lanthanide.


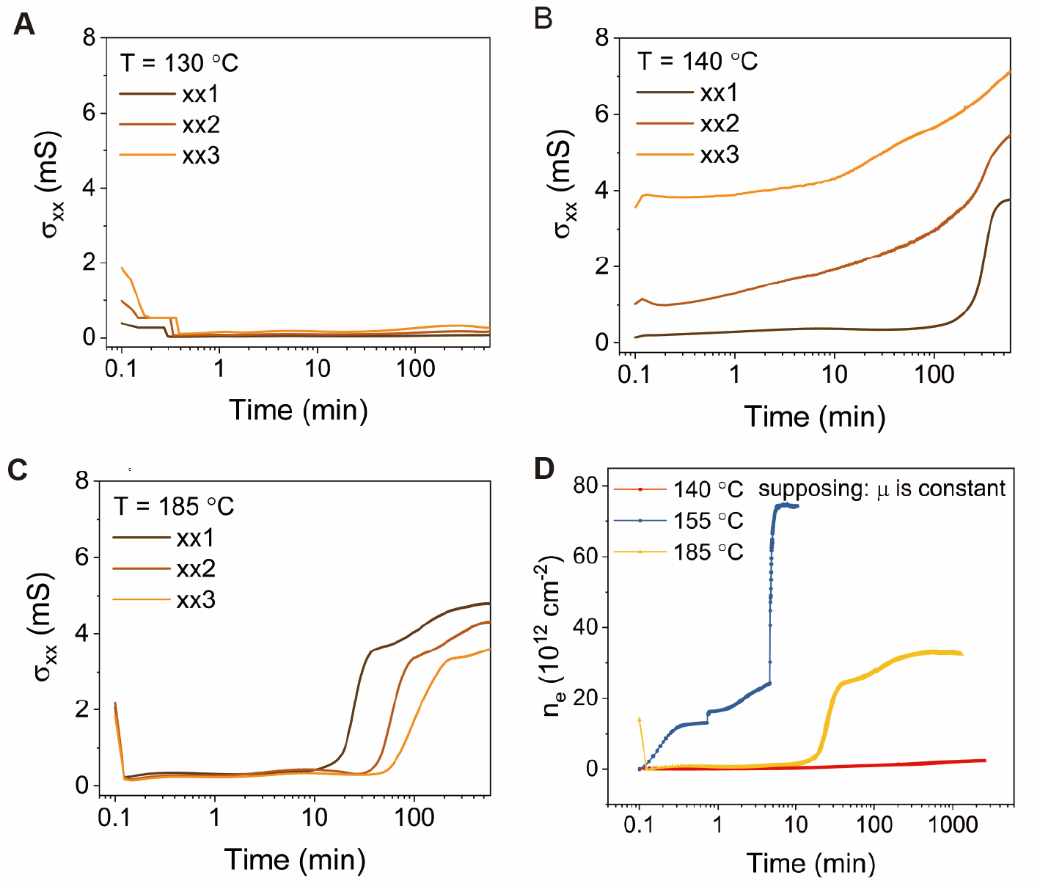


**fig. S7:** **Electrochemical intercalation of europium at different temperatures.** **A,** Time dependence of conductivity of BLG device 5 intercalated at 130 °C. **B,** Time dependence of conductivity of BLG device 6 intercalated at 140 °C. **C,** Time dependence of conductivity of BLG device 7 intercalated at 185 °C. **D,** time dependence of estimated density of electrons transferred from intercalants, assuming the electron mobility is a constant during the intercalation process.


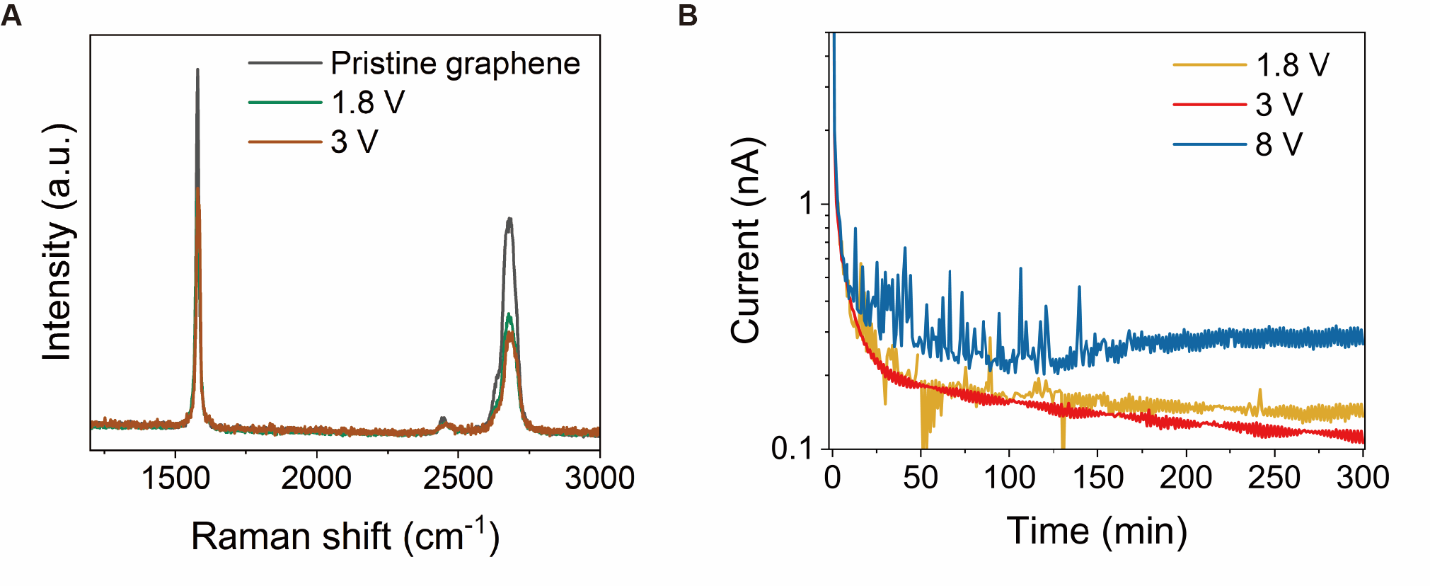


**fig. S8:** **Electrochemical intercalation at different in-plane bias (1.8 V and 3 V). A,** Raman spectrum of pristine graphene (black curve) and intercalated graphene (device 4) at 1.8 V (green curve) and 3 V (brown curve), separately. **B,** Time dependence of current flow between BLG (device 4) and counter electrode at T = 155 °C.

**Additional references:**

1. D.R. Lide, CRC Handbook of Chemistry and Physics: A Ready-reference Book of Chemical and Physical Data (CRC Press 1995).
2. C.G. Zoski, Handbook of electrochemistry (Elsevier 2006).
3. S. Fraga, J. Karwowski, K. Saxena, Handbook of atomic data (Elsevier 1976).
4. J.F. Shackelford et al, CRC materials science and engineering handbook (CRC press 2016).
5. L. Pauling, The nature of the chemical bond (Cornell University Press, 1960).
6. M. Petrović et al, The mechanism of caesium intercalation of graphene. Nature communications 4, 2772 (2013).
7. N. Yabuuchi et al, Research development on sodium-ion batteries. Chemical reviews 114, 11636-11682 (2014).
8. Z.L. Xu et al, A new high-voltage calcium intercalation host for ultra-stable and high-power calcium rechargeable batteries. Nature Communications 12, 3369 (2021).
9. T.D. Gregory, R.J. Hoffman, R.C. Winterton, Nonaqueous electrochemistry of magnesium: applications to energy storage. Journal of the Electrochemical Society 137, 775 (1990).
10. Y. Liang et al, Current status and future directions of multivalent metal-ion batteries. Nature Energy 5, 646-656 (2020).
11. X. Liu et al. Adsorption and growth morphology of rare-earth metals on graphene studied by ab initio calculations and scanning tunneling microscopy. Physical Review B 82, 245408 (2010).
12. A.C. Ferrari et al. Raman spectrum of graphene and graphene layers. Physical review letters 97, 187401 (2006).
13. P. Blake et al. Making graphene visible. Applied physics letters 91, 063124 (2007).
14. M.J. Smith, C.J.R. Silva, Conductivity studies of a polymer electrolyte based on europium trifluoromethanesulphonate. Solid State Ionics 58, 269-273 (1992).
15. A. Eckmann et al. Probing the nature of defects in graphene by Raman spectroscopy. Nano letters 12, 3925-3930 (2012).
16. E. Lorchat et al. Filtering the photoluminescence spectra of atomically thin semiconductors with graphene. Nature nanotechnology 15, 283-288 (2020).
17. W. Bao et al. Approaching the limits of transparency and conductivity in graphitic materials through lithium intercalation. Nature communications 5, 4224 (2014).
18. M. Bruna et al. Doping dependence of the Raman spectrum of defected graphene. ACS Nano 8, 7432-7441 (2014).
19. K. Pielichowski, K. Flejtuch, Non-oxidative thermal degradation of poly (ethylene oxide): kinetic and thermoanalytical study. Journal of Analytical and Applied Pyrolysis 73, 131-138 (2005).
20. Y. Liang et al, Current status and future directions of multivalent metal-ion batteries. Nature Energy 5, 646-656 (2020).
21. Y.M. Lin et al. Multicarrier transport in epitaxial multilayer graphene. Applied Physics Letters 97, 112107 (2010).
22. P. Giannozzi et al. Advanced capabilities for materials modelling with Quantum ESPRESSO. Journal of physics: Condensed matter 29, 465901 (2017).
23. J. Klimeš, D. R. Bowler, A. Michaelides, Van der Waals density functionals applied to solids. Physical Review B—Condensed Matter and Materials Physics, 83, 195131(2011).
